# Clinical effects of music therapy on menopausal symptoms

**DOI:** 10.1590/1806-9282.20240426

**Published:** 2024-07-19

**Authors:** Maria Eduarda da Macena Tenorio, Williames Matheus Malaquias da Silva, Brenda dos Santos Teixeira, Wendel Aguiar Carlini, Mylenne Alinne Falcão de Paiva, Johnnatas Mikael Lopes

**Affiliations:** 1 Universidade Federal do Vale do São Francisco – Paulo Afonso (BA), Brazil.

Dear Editor,

Over time, women begin to suffer from the interruption of their ovulatory cycles, the transition to the non-reproductive period, known as climacteric, with the last date of menstrual bleeding being defined as menopause^1^. During the climacteric period, each woman needs to be evaluated individually, so that her treatment is carried out appropriately, as the endocrine, psychological, and social factors lead to climacteric syndrome. The most common symptoms during the transitional phase are vasomotor symptoms, also known as hot flushes or hot flashes. In addition, women often experience mood changes, irritability, insomnia, urinary incontinence, as well as an increased risk of vaginal infections and osteoporosis^2^.

The study by Ugurlu et al., entitled "The effect of music on menopausal symptoms, sleep quality, and depression: a randomized controlled trial"^3^, presented us with a non-drug therapeutic approach for managing menopausal symptoms. However, only a statistical perspective was presented, with information on the clinical utility of the approach being absent.

Based on this gap, we developed a standardized clinical effect measure in order to understand the practical usefulness of music therapy in the management of menopause. Accepting that the measurements presented in tables 2 and 3 are means and standard deviations of the outcomes determined by the paired Student's t-test in some situations, we constructed our clinical effect measurements by estimating the unbalanced Cohen's d^4^ (Table 1).

**Table 1:** Measurement of clinical effects on somatic, psychological, and urogenital outcomes based on unbalanced Cohen’ d.

	x1	x2	SD1	SD2	x1-x2	SDm	N	√SD	Cohen's d
MRS total	12.40	16.00	6.51	4.12	-3.6	1738.255	59	5.427886	-0.66324
Somatic symptoms	4.97	5.87	2.13	1.69	-0.9	217.2531	59	1.918921	-0.46901
Psychological symptoms	4.57	7.16	3.41	2.71	-2.59	557.5379	59	3.074052	-0.84254
Urogenital symptoms	2.87	3.06	2.13	2.13	-0.19	267.6771	59	2.13	-0.0892
BDI	7.5	12.77	4.33	6.82	-5.27	1939.09	59	5.732882	-0.91926
PSQI	6.23	8.16	2.58	2.55	-1.93	388.1106	59	2.56479	-0.7525

x1: intervention group average; x2: mean of the control group; SD1: standard deviation of the intervention group; SD2: standard deviation of the control group; SDm: mean standard deviation; N: total participants minus the number of groups; √SD: square root of SDm; Cohen's d: (x1-x2)√SD.

The clinical effect on the general symptomatology of music therapy is considered moderate (d=0.66). However, when observing its dimensions, it is clear that music therapy is more effective in psychological symptoms, which are of great magnitude (d=0.84), the same being evidenced by the Beck depression index (d=0.91). On the other hand, the effects on somatic symptoms are of small magnitude (d=0.46). Urogenital symptoms confirmed the absence of significant differences between the groups with a trivial clinical effect (d=0.08).

Finally, sleep quality was also affected by the music therapy approach with great clinical implications (d=0.75). This effect probably occurs due to the strong relationship between psychological state and sleep regulation. Understanding these effects in a standardized way and with a clinical perspective reveals the usefulness of the music therapy approach as a therapeutic option in the management of psychobehavioral disorders during menopause.

Furthermore, it is worth highlighting that not using more elaborate statistical strategies such as Mixed Models reduces the understanding of the effect of individual variability on experiments, as each individual evolves at a different rate over the treatment period, which we call a random effect and this becomes necessary to be taken into account when analyzing data through methods called mixed models^5^. This is valid, as a higher concentration of low education in the control group is also observed in the data.
